# The dual role of HOP2 in mammalian meiotic homologous recombination

**DOI:** 10.1093/nar/gkt1234

**Published:** 2013-12-03

**Authors:** Roberto J. Pezza, Oleg N. Voloshin, Alexander A. Volodin, Kingsley A. Boateng, Marina A. Bellani, Alexander V. Mazin, R. Daniel Camerini-Otero

**Affiliations:** ^1^Oklahoma Medical Research Foundation, Oklahoma City, 73104 OK, USA, ^2^Department of Cell Biology, Oklahoma University Health Science Center, Oklahoma City, 73126 OK, USA, ^3^Genetics and Biochemistry Branch, National Institute of Diabetes, Digestive and Kidney Diseases, National Institutes of Health, Bethesda, 20892 MD, USA, ^4^Institute of Molecular Genetics of the Russian Academy of Sciences, 123182 Moscow, Russia, ^5^Biomedical Research Center, National Institute of Aging, Baltimore, 21224 MA, USA and ^6^Department of Biochemistry and Molecular Biology, Drexel University College of Medicine, Philadelphia, 19102 PA, USA

## Abstract

Deletion of *Hop2* in mice eliminates homologous chromosome synapsis and disrupts double-strand break (DSB) repair through homologous recombination. HOP2 *in vitro* shows two distinctive activities: when it is incorporated into a HOP2–MND1 complex it stimulates DMC1 and RAD51 recombination activities and the purified HOP2 alone is proficient in promoting strand invasion. We observed that a fraction of *Mnd1^−/−^* spermatocytes, which express HOP2 but apparently have inactive DMC1 and RAD51 due to lack of the HOP2–MND1 complex, exhibits a high level of chromosome synapsis and that most DSBs in these spermatocytes are repaired. This suggests that DSB repair catalyzed solely by HOP2 supports homologous chromosome pairing and synapsis. In addition, we show that *in vitro* HOP2 promotes the co-aggregation of ssDNA with duplex DNA, binds to ssDNA leading to unstacking of the bases, and promotes the formation of a three-strand synaptic intermediate. However, HOP2 shows distinctive mechanistic signatures as a recombinase. Namely, HOP2-mediated strand exchange does not require ATP and, in contrast to DMC1, joint molecules formed by HOP2 are more sensitive to mismatches and are efficiently dissociated by RAD54. We propose that HOP2 may act as a recombinase with specific functions in meiosis.

## INTRODUCTION

Double-strand breaks (DSBs) are a severe type of chromosomal DNA damage. They arise spontaneously through exogenous and endogenous causes such as radiation or free radicals and, interestingly, also occur during the course of the developmental program of meiosis. Homologous recombination (HR) is the only process that assures error-free repair of most DSBs (1,2). In meiosis HR also provides the associations between homologous chromosomes that are required for their proper segregation (3,4). This has a direct impact on faithful haploidization of a genome and avoidance of aneuploidy. Indeed, failure of proper homologous chromosome segregation leads to severe aneuploidy-related birth defects such as Down, Klinefelter, Edwards and Turner syndromes (5). Critical functions in HR are provided by the ubiquitous RAD51 and the meiosis-specific DMC1 recombinases. These enzymes repair DSB by promoting the invasion of intact double-stranded DNA (dsDNA) by single-stranded (ssDNA) ends (6). It is currently accepted that strand invasion intermediates proceed by one of two distinct pathways (7–9). They can dissociate after extension of the invading 3′-end with subsequent rejoining of the broken chromosome by synthesis-dependent strand annealing pathway (SDSA) to generate non-crossover (NCO). Alternatively, they proceed via the double-strand break repair mechanism (DSBR) (10,11), generating CO (7,8,11). DMC1 and RAD51 recombinases cannot function alone and require accessory proteins whose functions are poorly understood. Among them are HOP2 and MND1, key accessory proteins necessary for normal progression of HR. These two proteins function through their interaction with DMC1 and RAD51 (6,12–22). We and others have previously shown that *in vitro* the HOP2–MND1 complex increases the stability of the DMC1/RAD51-ssDNA filament found on resected DSBs and promotes capture of potential partner chromosomes to facilitate the search for homology and generation of joint molecules by DMC1 and RAD51 through strand invasion (21–23). Here, we present evidence that HOP2 *in vivo* can work alone as a *bona fide* recombinase in addition to stimulating the activities of DMC1 and RAD51 as a part of the HOP2–MND1 heterodimer. We show that despite the uniqueness of its recombination pathway, HOP2 *in vitro* possesses mechanistic signatures characteristic of the mammalian RecA-like recombinases DMC1 and RAD51.

## MATERIALS AND METHODS

Experiments conformed to relevant regulatory standards and were approved by the IACUC (Institutional Animal Care and Use Committee).

### Generation of *Mnd1*-deficient mice

The mouse embryonic stem (ES) cell line RRS590 containing a gene trap insertion in *Mnd1* was obtained from BayGenomics (baygenomics.ucsf.edu/). The gene-trapping vector used to create this line, pGT0Lxf, was designed to create an in-frame fusion between the 5′-exons of the trapped gene and a reporter, β*geo* (a fusion of β-galactosidase and neomycin phosphotransferase II). The vector was inserted into intron 5 of *Mnd1*, resulting in the generation of a transcript containing exons 1–5 of *Mnd1* and β*geo.* To precisely identify the insertion site within intron 5, PCR reactions were performed using one primer hybridizing at the 5′-end of the gene trap vector and the complementary primer obtained by 5′-RACE PCR from the mouse ES cell clone RRS590. The PCR product was sequenced, revealing that the insertion site was 926 bp into intron 5 (Supplementary Figure S1). RRS590 ES cells were injected into C57BL/6 blastocysts to create chimeric mice, which were bred with C57BL/6 to generate heterozygous (+/−) lines that were variously intercrossed to other mutant lines.

### Surface spreading of meiotic chromosomes and immunocytochemistry

The methods used for surface spreading of spermatocytes and immunolabeling of meiotic chromosomes have been described (19,24). Sources and dilutions of primary antibodies used are as follows: rabbit anti-HOP2–MND1 antibody raised against the full-length HOP2–MND1 complex was used at a dilution of 1:300. Mouse anti-SYCP3 (Novus), 1:400; rabbit anti-SYCP1 (Novus), 1:200; Mouse CREST antisera (a gift from B.R. Brinkley), 1:200; rabbit anti-RAD51 and rabbit anti-DMC1 (Santa Cruz Biotechnology), 1:80; mouse RPA (Novus), 1:150; rabbit anti-biotinylated-phosphorylated H2AX (γH2AX) at serine 139 (Upstate Biotechnology), 1:500; mouse anti-MLH1 (BD Pharmingen, BD Biosciences), 1:30. All secondary antibodies were from Jackson IR laboratories and were used at a dilution of 1:200. Slides were subsequently counterstained for 3 min with 2 µg/ml DAPI containing Vectashield mounting solution (Vector Laboratories) and sealed with nail varnish. All images were acquired using a 40× objective oil immersion lens. We use Axiovision SE 64 (Carl Zeiss, Inc.) for imaging acquisition and processing.

### Proteins, DNAs and oligonucleotides

Mouse HOP2 and MND1 proteins and the HOP2–MND1 complex were purified as described previously (18). Human DMC1 was purified according to published protocols (25). RAD54 was purified as described in previous protocol (26). RecA was purified as described (27). pUC19 (except for that used to form D-loops) and ϕX174 were purchased from New England BioLabs. Oligonucleotides, some of which were tagged with a biotin or fluorescent dies residues (as specified in Supplementary Table S1), were obtained from MWG Biotech and IDT, Inc. The ssDNA oligonucleotides used in this study were purified by denaturing PAGE. The supercoiled pUC19 dsDNA used in D-loop assay was purified with Hi-Speed Plasmid Maxi Kit (Qiagen) followed by CsCl banding. The concentration of ssDNA and dsDNA were determined by absorbance at 260 nm, using 36 µg and 50 µg/ml/A260, respectively, as conversion factors and are expressed as molar concentrations of nucleotides or base pairs for ssDNA or dsDNA, respectively.

### dsDNA capture

dsDNA capture was performed essentially as previously described (21). Briefly, HOP2 (2, 5 and 10 µM) and DMC1 (10 µM) were preincubated with biotinylated oligonucleotide ssDNA (#1, Supplementary Table S1) prebound to streptavidin coated agarose beads (60 µM nucleotides) in standard binding buffer (20 mM Tris–HCl pH 7.4, 70 mM NaCl, 2.5 mM MgCl_2_, 1 mM DTT, 2 mM ATP, 7.5 mM creatine phosphate, 30 U/ml creatine kinase) for 5 min at 37°C. HOP2–MND1 (0.4 µM) was added to reactions containing DMC1. An amount of 75 µM pUC19 DNA was then added and reactions were incubated for an additional 10 min. After completion of the reaction, beads were washed twice with standard buffer and resuspended in 25 µl of the same buffer, followed by deproteinization (0.5% w/v SDS, 1 mg/ml proteinase K) for 20 min at 37°C. Products were resolved on 1% agarose gels and the bands were visualized by ethidium-bromide staining, quantitated using a BAS 2500 Bio-Imaging Analysis System (Fuji Medical System). Pixel counts were used as a measure of total dsDNA captured.

### Chemical probing of ssDNA–HOP2 complexes

Partial duplex DNA obtained by annealing of 68-mer 5′-^32^P-labeled synthetic oligonucleotide and its 23-mer complement (oligonucleotides #2 and #3, Supplementary Table S1) was used as a substrate for DNA binding. HOP2 reaction buffer consisted of 25 mM Tris acetate, 2 mM Mg acetate, pH 7.4. For RecA and DMC1 the same reaction mixture was supplemented with 0.1 mM ATPγS and 0.8 mM AMPPNP, respectively. Treatment with KMnO_4_ and subsequent procedures were performed as described in details in http://www.nature.com/protocolexchange/protocols/1908. Because the extent of KMnO_4_ modification is very sensitive to composition of the reaction buffer we performed a mock treatment of the DNA substrate in identical buffer conditions for each protein.

### Fluorometric assay of synaptic complex formation

The 3 µM 83-mer oligonucleotide (28) (#4, Supplementary Table S1) was preincubated with 1.5 µM HOP2 or 1 µM DMC1 in 25 mM Tris–HCl pH 7.4, 0.5 mM DTT, 100 µg/ml BSA and 5 mM MgCl_2_ at 37°C for 5 min. G16 duplex (#5/6, Supplementary Table S1) was then added at a final concentration of 6 µM and the reaction was continued at 37°C. Fluorescence resonance energy transfer (FRET) was measured as described previously (28–30). For pairing reactions, the 3′-end of the ssDNA oligonucleotide was labeled with fluorescein, and the 5′-end of the complementary strand in the duplex oligonucleotide was labeled with rhodamine. Quenching of fluorescein emission as a result of pairing was measured at 525 nm upon excitation at 493 nm with a SLM 8000C spectrofluorometer (ISS) at 2 s intervals.

### Preparation of native D-loops and their dissociation by RAD54

Preparation of native D-loops and their dissociation by Rad54 was performed essentially as previously described (31). Tailed ^32^P-labeled DNA was formed by annealing equimolar amounts of oligonucleotides (#7 and #8, Supplementary Table S1) and purified in native polyacrylamide gels. 14.26 nM DNA substrate was preincubated with DMC1 (1 µM) or RAD51 (3 µM) proteins in 15 µl of the buffer containing 25 mM Tris acetate, pH 7.5, 2.5 mM Mg acetate, 1 mM ATP, 2 mM DTT (40 mM for HOP2), 100 µg/ml BSA, 20 mM phosphocreatine and 30 U/ml creatine phosphokinase for 10 min at 37°C. HOP2 (5 µM) was incubated with both tailed oligonucletide and with pUC19 dsDNA. In the DMC1 and RAD51 reaction HOP2–MND1 (0.2 µM) and ammonium sulfate (50 mM) were included and incubated for an additional 10 min at 37°C, after which D-loop formation was initiated by addition of pUC19 plasmid (18 µM base pairs) followed by a 10-min incubation. Dissociation of protein-coated D-loops was initiated by addition of RAD54 (at indicated concentrations) and carried out for 3 min at the indicated times for the time course experiment. Reactions were stopped by addition of 0.5% SDS and 1 mg/ml proteinase K followed by incubation for 10 min at 37°C. Products were resolved on a 1% agarose gel; the gel was dried on DEAE paper and analyzed using a BAS 2500 Bio-imaging Analysis System (Fuji Medical System).

### Strand exchange reactions

Strand-exchange reactions were performed as described (32) with slight modifications. Incubation time was 1.5 h for DMC1 and 2 h for HOP2 at 37°C; protein to DNA ratio was 1 monomer/2 bases of ssDNA substrate for DMC1 and 1 monomer/3 bases for HOP2; 2 mM CaCl_2_ for DMC1 and 6 mM CaCl_2_ for HOP2; 1 mM ATP in DMC1 promoted reaction. Oligonucleotides (#9–#16) used in this assay are described in Supplementary Table S1.

## RESULTS

### Homologous chromosome synapsis in *Mnd1^−^^/^*^−^ spermatocytes

**Figure 1.**
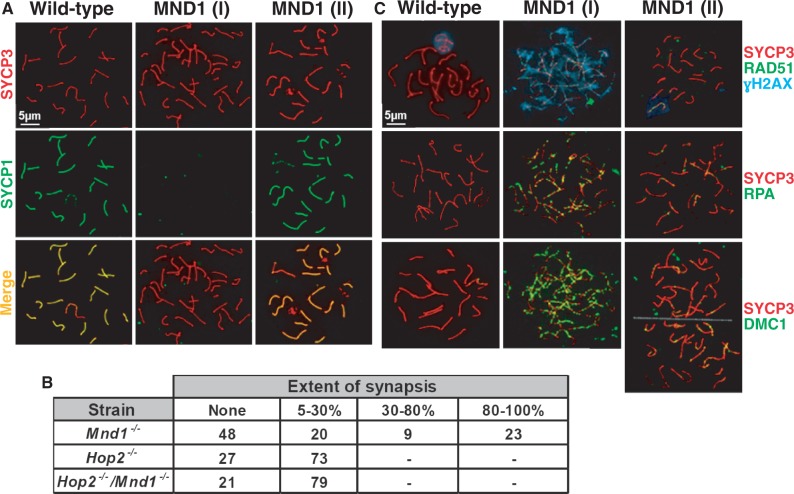
Synaptonemal complex assembly defects in prophase I meiocytes from *Mnd1^−/−^* mice. All images were taken under the same magnification, however the 5 µm scale bar is shown only for wild-type spermatocytes. (**A**) Co-immunostaining of SYCP3 and SYCP1 in wild-type and *Mnd1^−/−^* pachytene spermatocytes. Under MND1 (I) is shown a representative spermatocytes with fully developed axial elements but completely asynapsed chromosomes. MND1 (II) shows an example of spermatocyte with extensive chromosome synapsis. Only examples of the most contrasting types of spermatocytes with either absence or total chromosome synapsis are shown. (**B**) In contrast to *Hop2^−/−^* or *Hop2^−/−^/Mnd1^−/−^* meiocytes, fractions of spermatocytes from *Mnd1^−/−^* mice show extensive chromosome synapsis. Spermatocytes are divided into four groups according to the extent of chromosomal synapsis. A total of 100 arrested spermatocytes were randomly picked and counted for each group. (**C**) Localization of γH2AX, RAD51 and DMC1 in spermatocytes of *Mnd1^−/−^* mice. (I) and (II) represent the most contrasting fractions of *Mnd1^−/−^* spermatocytes as in (A). Wild-type spermatocytes are shown for comparison.

We have previously shown that association with MND1 provokes changes in HOP2 that are responsible not only for abrogating the intrinsic recombinase activity of HOP2 but also for generating a new molecular interface essential for stimulating the recombinational activities of DMC1 and RAD51 (18,20). Consequently, we reasoned that knocking out MND1 will render DMC1 and RAD51 inactive and make the entire pool of HOP2 available to promote recombination. To test this hypothesis we generated mice with a gene trap-disrupted allele, *Mnd1*^RRS590^ (Supplementary Figure S1). The gene was disrupted by the insertion of the β-galactosidase-neomycin cassette in intron 5 of *Mnd1* (Supplementary Figure S1A). Western blot analysis using polyclonal affinity-purified antibodies raised against recombinant HOP2–MND1 show no signal of full-length MND1 in testis cell extracts (Supplementary Figure S1B). In agreement, RT–PCR analysis using a pairs of oligonucleotides specific for *Mnd1* exon boundaries 7–8 show that *Mnd1^−/−^* testes do not express any of the trapped exons (Supplementary Figure S1C). We also look for a possible partial product of *Mnd1* expression (exons 1–5) fused with β-galactosidase and neomycin. We observed no signal for this protein fusion when we performed Western blots in *Mnd1^−/−^* testis cell extracts using antibodies specific for β-galactosidase and those raised against recombinant HOP2–MND1 (data not shown). Thus, our *Mnd1* knockout mice do not express any part of the MND1 protein. Six-month-old knockout mice showed defects in male and female gametogenesis (Supplementary Figure S2A–C). These results show that MND1 plays an important role in mouse meiosis. We observed a lack of advanced spermatogenic cells, most likely as a consequence of primary spermatocytes undergoing apoptosis (Supplementary Figure S2B). To understand the origins of the meiotic defects in the *Mnd1^−/−^* mice, we analyzed the progression of spermatogenesis through prophase I using immunostaining of spermatocyte chromosome spreads (24). Co-immunostaining for the synaptonemal complex proteins SYCP3 and SYCP1 (used to analyze sister chromatid cohesion and homologous chromosome synapsis, respectively) revealed that meiosis appears to progress normally through leptotene and zygotene. Interestingly, in later stages we observed different populations of spermatocytes with profound differences in the extent of homologous chromosomes synapsis and DNA repair. For quantification, we divided these *Mnd1^−/−^* spermatocytes into discreet categories. The first group [MND1 (I)] displayed very limited chromosome synapsis with normal sister chromatid cohesion (Figure 1A and B). One reasonable explanation for this is that the absence of the HOP2–MND1 heterodimer in these cells leads to deficiency in strand invasion catalyzed by DMC1 and RAD51 (see below) and, as a consequence, homologous chromosomes fails to synapse. We also observed a population of spermatocytes with intermediate levels of synapsis ranging from 5% to 30% (Figure 1B). Notably, fractions of *Mnd1^−/−^* spermatocytes [MND1 (II)] in an apparent zygotene/pachytene like stage exhibited extensive synapsis (32% of total scored cells showed 30–100% of synapsis) (Figure 1A and B). The extent of homologous chromosomes synapsis in these meiocytes is remarkably higher than in *Hop2^−/−^* and *Hop2^−/−^*/*Mnd1^−/−^* (Figure 1B). We also immunostained wild-type and *Mnd1^−/−^* spermatocytes with CREST and SYCP3 (Supplementary Figure S3A). Our results suggest that the synapsis observed in MND1 (II) spermatocytes is homologous. Because *Hop2*^−/−^ spermatocytes show a very limited extent of pairing and synapsis (17) (Figure 1B), the results obtained with *Mnd1^−/−^* spermatocytes suggest that HOP2 by itself is sufficient to support advanced levels of homologous chromosomes association. We propose that the observed phenotype is likely due to the ability of HOP2 to catalyze strand invasion in the context of both *Mnd1^−/−^* [(15,17,18,20) and this work] and wild-type (see below) spermatocytes.

### HOP2 promotes repair of DSBs *in vivo*

Since chromosome synapsis and HR are co-dependent processes [(33,34) and references within], we asked whether the striking differences observed in homologous chromosomes synapsis for the subsets of *Mnd1^−/−^* spermatocytes [(MND1 (I) and MND1 (II)] is related to their ability to repair DSBs. We assessed localization patterns of several proteins that are cytological indicators of HR progression in meiotic prophase I. Recombinational repair in leptotene-zygotene *Mnd1^−/−^* spermatocytes appeared to initiate normally as is evident by the presence of γ-H2AX staining and RAD51 and DMC1 foci formation. Thus, in all the early meiocytes analyzed, DSBs were formed and apparently processed into 5′-resected ssDNA. However, for the *Mnd1^−/−^* zygotene-like spermatocytes exhibiting very limited or no synapsis [MND1 (I)], defects in DSB repair became apparent through the persistence of γH2AX staining. In these spermatocytes, RAD51, RPA, DMC1 and NBS1 staining are abundant and localize all over the chromosome core (Figure 1C and Supplementary Figure S3B). Thus DSBs are created and processed followed by DMC1 and RAD51 loading on recessed DNA ends. However, in the absence of the HOP2–MND1 complex, initiation of strand invasion cannot occur. These observations are in agreement with our previous *in vitro* results showing that the HOP2–MND1 complex is an essential factor required for efficient DNA repair mediated by DMC1 and RAD51 (18,20–23).

Similar analyses of *Mnd1^−/−^* zygotene-like spermatocytes exhibiting complete chromosomal synapsis [MND1 (II)] revealed that few RAD51, DMC1 and RPA foci are detectable and that the γ-H2AX staining disappears except in the sex body region (Figure 1C). This phenotype resembles that of wild-type spermatocytes and suggests that in the absence of MND1 (which renders DMC1 and RAD51 inactive), HOP2 alone may catalyze repair of DNA. This recombinational function of HOP2 is consistent with our previous biochemical data showing that purified HOP2 can efficiently form D-loops *in vitro* (18,20).

### In the absence of MND1 DSBs are repaired but no COs are observed

**Figure 2.**
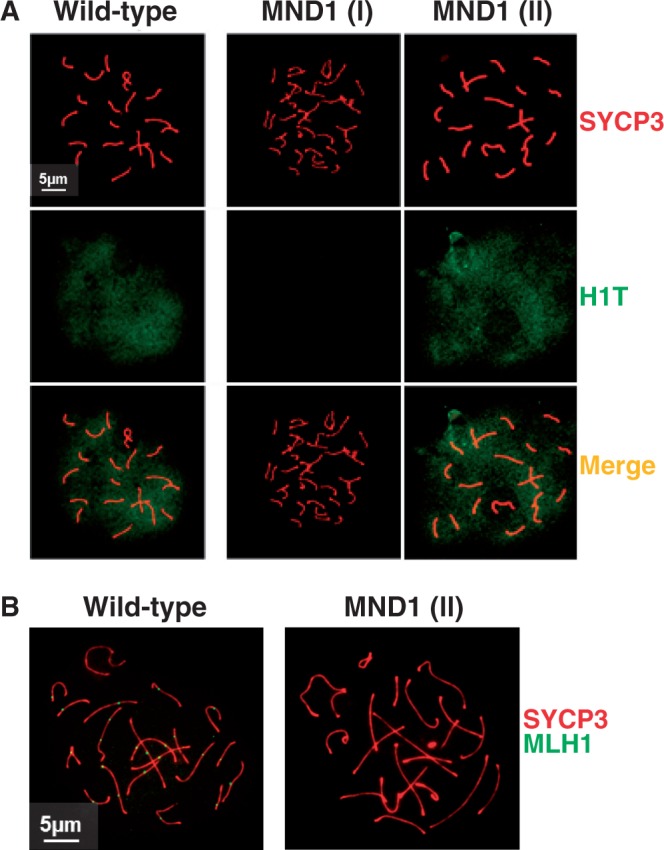
Recombination events marked by MLH1 are deficient in MND1 (II) type spermatocytes. (**A**) Example of a wild-type spermatocyte and arrested *Mnd1^−/−^* spermatocytes showing no synapsis [MND1 (I)] and complete synapsis [MND1 (II)] co-immunostained with SYCP3 and H1T. (**B**) Example of a wild-type spermatocyte and an arrested *Mnd1^−/−^* spermatocyte showing complete synapsis [MND1 (II)] co-immunostained with SYCP3 and MLH1. Note the absence of MLH1 staining in the MND1 (II) spermatocyte. The magnification bar represents 5 µm and corresponds to all images but shown only in one panel.

We asked whether in the group of spermatocytes exhibiting complete chromosomal synapsis [MND1 (II)], a fraction of recombination intermediates were resolved as CO. We scored a total of 100 *Mnd1^−/−^* spermatocytes exhibiting total chromosome synapsis. We observed that 60% of cells were in advanced pachytene-like stage (evaluated by histone H1T positive staining, Figure 2A), however only 6% of these cells showed background numbers of MLH1 foci (3.0 ± 1.1 foci per cell) (representing 10% of cells with positive staining for H1T) (Figure 2B). In sum, recombination events marked by MLH1 in MND1 (II) type spermatocytes that are destined to be resolved as COs are remarkably deficient.

### HOP2 may work as a recombinase independent of DMC1/RAD51 and MND1

We observed that the level of HOP2 protein in spermatocytes of *Mnd1^−/−^* mice is significantly reduced compared to wild-type (Supplementary Figure S1B and C). We then tested the possibility that the variable extent of homologous chromosomes synapsis depends on the differential expression of *Hop2*. We performed a combination of RNA FISH and immunostaining on spermatocyte preparations that preserve nuclear structure (Supplementary Figure S4). For quantification purposes, based on SYCP1 staining we divided *Mnd1^−/−^* spermatocytes in three categories, no synapsis, 5–60% synapsis and 60–100% synapsis. We observed that 33% of *Hop2^−/−^* cells showed an RNA FISH signal for the *Hop2* transcript; this fraction represents the background signal. A low percentage of cells with unpaired and partially synapsed chromosomes showed signal for *Hop2* transcript (30% and 33%, respectively). However, similar to wild-type (78% of zygotene-pachytene cells with *Hop2* RNA FISH signal) the fraction of *Mnd1^−/−^* spermatocytes with extensive chromosome synapsis (60–100%) contained 65% of cells that were positive for the *Hop2* transcript. These results provide additional evidence supporting the hypothesis that HOP2 by itself may work as a recombinase *in vivo*.

If in wild-type spermatocytes HOP2 has a dual role in meiosis we expect that a fraction of HOP2 will not be incorporated into the HOP2–MND1 complex and will be freely available to catalyze recombination. To test this we measured the relative concentration of HOP2 and MND1 in cell-sorted enriched fractions of wild-type spermatocytes of adult mice (Supplementary Figure S5). We found that the amounts of HOP2 and MND1 peaked at the zygotene-late-pachytene stage with a 3:1 HOP2:MND1 ratio. Thus our results confirm the presence of free MND1-unbound HOP2 in wild-type meiocytes and support the possibility that HOP2 functions as a DMC1/RAD51 independent recombinase *in vivo*.

### HOP2 possesses fundamental homolog-recognition properties

**Figure 3.**
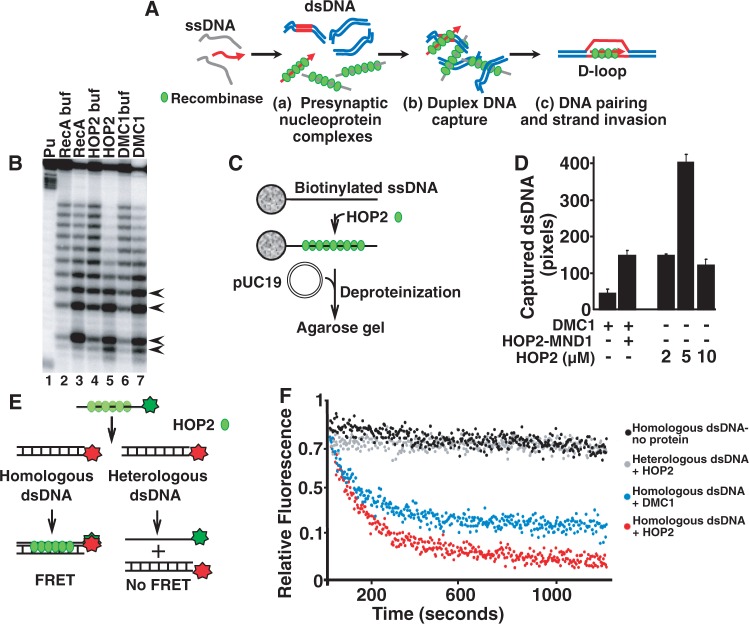
The mechanism of recombination mediated by HOP2. (**A**) Formation of joint molecules promoted by HOP2. (Panel a) The presynaptic polymerization of HOP2 protein on ssDNA. (Panel b) Conjunction of ssDNA and dsDNA without homologous alignment. (Panel c) Homologous DNA pairing and strand invasion. Deproteinization of the complex in panel (c) in the context of a supercoiled target duplex DNA results in a stable D-loop. (**B**) HOP2 unstacks bases upon binding to ssDNA. Unstacking is manifested as increased reactivity of the thymine residues to potassium permanganate (arrows). Lane 1 contains a ladder generated by cleavage at purine bases (**C**) Experimental outline for DNA capture assay. Biotinylated DNA, immobilized on streptavidin–agarose beads, was used as a binding substrate for HOP2 protein. After incubation with pUC19 dsDNA, nucleoprotein complexes were separated from unbound proteins and dsDNA by centrifugation, and products were deproteinized before analysis by gel electrophoresis. (**D**) Quantitation of captured DNA as determined by intensity of ethidium bromide fluorescence. (**E**) Schematic of the synaptic complex assay. Fluorescein and rhodamine are represented in green and red, respectively. (**F**) Synaptic complex formation promoted by HOP2 and DMC1 with homologous and heterologous DNA. Synaptic complexes for homologous dsDNA in the absence of protein and DMC1 with homologous DNA are also shown.

We have previously shown that HOP2 is proficient in promoting strand invasion *in vitro* with an efficiency close to that for classical recombinases such as bacterial RecA and meiotic DMC1 (18,20). To unravel the molecular mechanism by which HOP2 recognizes DNA homology and catalyzes strand invasion we studied *in vitro* biochemical properties that are considered to be hallmarks of homology recognition in the bacterial RecA and the eukaryotic recombinases DMC1 and RAD51. For this purpose, it is useful to distinguish at least three consecutive steps that have been revealed through the study of RecA-mediated strand invasion. The first step is presynaptic nucleoprotein complex formation; the second is conjoining of DNAs (duplex DNA capture), which is not mediated by homologous interactions but is a prerequisite for the homologous pairing in the next step (Figure 3A) The third, homology-dependent step, is formation of the first product of homologous pairing, the synaptic complex (Figure 3A) [reviewed in (6); (18,20)]. In this study, we show that HOP2 is able to catalyze all these three critical steps required for homologous pairing.

DNA base unstacking upon binding of a recombinase is a hallmark of HR (35–37). Using chemical footprinting we observed that similarly to the classical recombinases RecA and DMC1, HOP2 unstacks bases in the complex with ssDNA. Extension of DNA within the nucleoprotein complex results in hypermodification of the thymine bases by potassium permanganate which is manifested as conversion of full-size DNA strand into shorter fragment visible at the bottom of the gel (lanes 3, 5 and 7 in Figure 3B).

Prior to the strand invasion reaction, the nucleoprotein complex formed by the recombinases and bound ssDNA must recruit the duplex DNA molecule. Using a previously published DNA capture assay (21) involving the immobilization of biotinylated ssDNA on streptavidin–agarose beads as a binding substrate for HOP2, we found that, like DMC1, purified recombinant HOP2 promotes the co-aggregation of ssDNA with duplex DNA (Figure 3C and D).

Before strand invasion (D-loop formation), RecA promotes homologous alignment of ssDNA and dsDNA molecules (38). In this reaction, a presynaptic nucleoprotein complex of ssDNA and recombination proteins is allowed to find its homologous target embedded in an otherwise heterologous DNA, and the product is the synaptic complex (Figure 3A, panel c). We studied synaptic complex formation using an assay based on FRET (Figure 3E). This assay is non-disruptive and allows observation of the reaction in real time (28–30). We used a ssDNA oligonucleotide (negative strand) labeled with fluorescein and homologous dsDNA in which only the complementary strand was labeled with rhodamine (Supplementary Table S1). During the synaptic complex reaction, a three-strand intermediate forms, bringing fluorescein and rhodamine into close proximity resulting in FRET in which fluorescein excited by light transfers energy to rhodamine and is quenched. A decrease in the sensitized emission shows that the dyes have come into proximity and detects the formation of the first step in homology-dependent interaction of the DNA strands. We observed the formation of the synaptic complex with homologous DNA substrates and HOP2 (or DMC1 as a control), whereas no DNA pairing (no significant fluorescence quenching) was observed with heterologous DNAs (Figure 3F). We note that the extent of synaptic complex formation catalyzed by HOP2 versus DMC1 may reflect slightly different conditions in the assay for these two recombinases, i.e. protein concentration. Taken together, our results show that HOP2 shares fundamental ‘homology-recognition’ properties with the RecA homologs (29,30,39).

### D-loops catalyzed by HOP2 are substantially more sensitive to dissociation by RAD54 than those formed by DMC1

**Figure 4.**
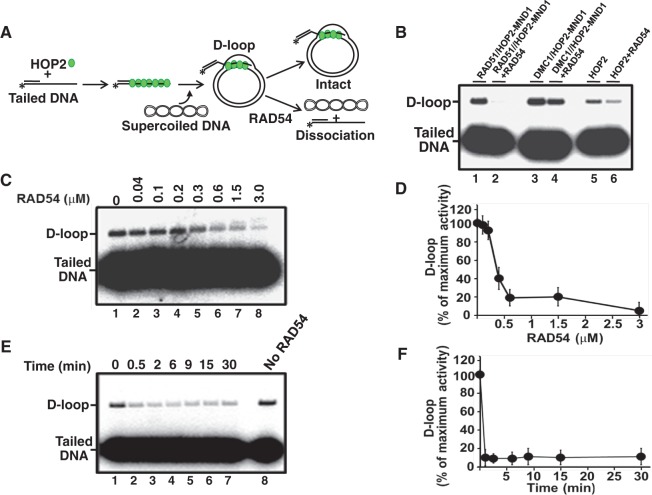
RAD54 dissociates native D-loops formed by HOP2. (**A**) Experimental scheme. Asterisk denotes ^32^P-label. (**B**) Analyses of D-loops in a 1% agarose gel. D-loops formed by RAD51/HOP2–MND1 (lane 1), DMC1/HOP2–MND1 (lane 3) and HOP2 (lane 5) were tested for dissociation by adding purified 0.32 µM RAD54 for DMC1/RAD51 (lanes 2 and 4. respectively) and 0.64 µM for HOP2 (lane 6) for 9 min at 37°C. (C) Dissociation of HOP2-coated D-loops as a function of RAD54 concentration. Lanes 1–8 show remaining D-loop after treatment with 0, 0.04, 0.1, 0.2, 0.32, 0.4, 1.5 and 3.0 µM RAD54. (**D**) Quantitation of radioactive signals corresponding to remaining D-loops shown in panel c. (**E**) Kinetics of dissociation of native HOP2-mediated D-loops by 0.64 µM RAD54. Lanes 1–7 show remaining D-loop after 0, 0.5, 2, 6, 9, 15 and 30 min after addition of RAD54. Lane 8 shows D-loop in absence of RAD54 after 30 min of incubation. One hundred percent represents maximum of activity observed. (**F**) Results of the radioactive signal quantification of the gel in (E) were plotted.

We previously reported, and confirmed here, that RAD54 may control a CO/NCO decision by differentially dissociating native D-loops formed by RAD51 but not DMC1 [(31) and Figure 4A and B]. The absence of CO products in the fraction of *Mnd1^−/−^* spermatocytes with extensive chromosome pairing [MND1 (II)] suggested that RAD54 might dissociate strand invasion products catalyzed by HOP2 and thus lead to the formation of intermediates resolved through the SDSA pathway. We tested this hypothesis by challenging native HOP2-mediated D-loops with the RAD54 protein. DMC1 and RAD51 were used as controls and are consistent with published data (31). Under our experimental conditions, HOP2 (26% of supercoiled dsDNA in D-loop), DMC1 (38%) and RAD51 (31%) showed comparable initial yields of protein decorated D-loops (Figure 4B). We observed that both HOP2 and RAD51native D-loops were efficiently dissociated by treatment with RAD54. In contrast, DMC1 native D-loops were insensitive to RAD54 treatment (Figure 4B–F). These results suggest that native D-loops formed by HOP2 are more sensitive than DMC1 to RAD54 dissociation.

### Reduced tolerance to mismatches of HOP2-mediated strand exchange

**Figure 5.**
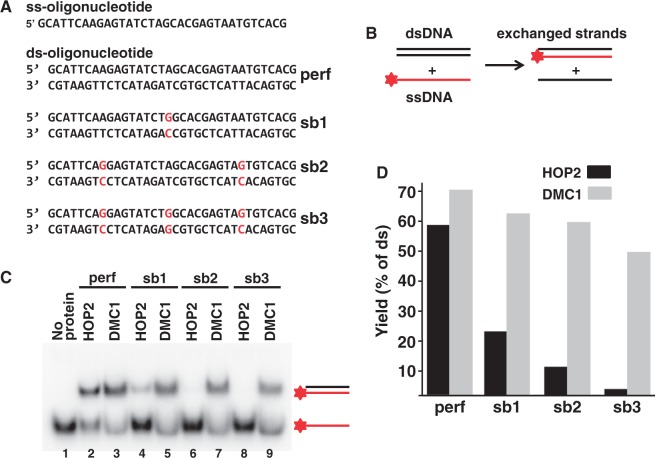
The tolerance to mismatches determines extent of the strand exchange reaction mediated by HOP2 and DMC1. (**A**) oligonucleotide substrates: dsDNA-substrate with perfect homology (perf) to the ssDNA-oligonucleotide and containing 1, 2 and 3 bp substitutions (sb1, sb2 and sb3, respectively; the substituted base pairs are shown in red). (**B**) The scheme of the strand exchange reaction. (**C**) Electrophoresis of the strand exchange products. The reaction with different dsDNA-oligonucleotide substrates (for designations see panel A) was promoted by HOP2 (lanes 2, 4, 6 and 8) or DMC1 (lanes 3, 5, 7 and 9) proteins. Lane 1, zero point control (components for HOP2 promoted strand exchange reaction between substrates with perfect homology were mixed at 0°C, deproteinized with addition of SDS and electrophoresed). (**D**) Quantification of the fluorescent signal corresponding to dsDNA oligonucleotide of the gel in (C) was plotted.

The discrimination of homology from heterology by a recombinase during strand exchange (30,40,41) may be used to distinguish HOP2 from other recombinases. We tested the ability of HOP2 and DMC1 to discriminate DNA substrates that contain mismatches using a previously validated protocol employing short oligonucleotides (32). While both recombinases were equally proficient in processing DNA substrates of identical sequence (oligonucleotide with perfect sequence), HOP2 exhibited a significant decrease in strand exchange with oligonucleotides containing mismatches. Notably, in sharp contrast to DMC1 only a single mismatch decreased HOP2 activity by ∼50% (Figure 5).

In sum, we show that HOP2 is biochemically distinct from DMC1 with respect to tolerance to mismatches and resistance to RAD54 dissolution. This suggests that HOP2 may be involved in repairing a unique subset of DSBs through a mechanism of recombination that defines a different pathway of DNA repair.

## DISCUSSION

### The function of MND1 in mouse meiosis

The *Mnd1* gene was first identified in *Saccharomyces cerevisiae* in a screen for genes with meiotic specific expression (42). The *S. cerevisiae Mnd1* null mutant shows defects in nuclear division, meiotic recombination and repair of DSBs (12,43,44). Indeed, cells initiate recombination but do not form heteroduplex DNA or a double Holliday junction suggesting that Mnd1 is probably involved in strand invasion. Accordingly, deletion of *mcp7*, a *Schizosaccharomyces pombe* ortholog of Mnd1 (45), and *Arabidopsis thaliana* Mnd1 genes (46–48), result in meiosis arrest and partial or total sterility.

The genetic interactions of *Mnd1* with *Hop2* and the *Dmc1* appear to be particularly important, as in all the genomes examined to date *Mnd1* and *Hop2* are found only in those organisms that have *Dmc1*. Although the function of *Mnd1* is apparently evolutionarily conserved, the cellular role of mammalian MND1 has to be yet elucidated. In this work we describe for the first time the phenotype of the *Mnd1^−/−^* mice. We show that this gene is essential for progression of normal meiosis and mouse fertility. Using cytological methods we determine that spermatocyte development arrest occurs at mid stages of meiosis I prophase with failure in both DNA repair and homologous chromosome synapsis.

Previous *in vitro* studies using purified protein proposed a possible mechanism of action for MND1 (18,20,23). We observed that the interaction of HOP2 with MND1 downregulates the strand assimilation activity of HOP2 (18,20). Furthermore, we observed that HOP2–MND1 binds DMC1 with a significantly increased affinity compared with isolated MND1 and HOP2, and only the HOP2–MND1 heterocomplex, but not the individual proteins, significantly stimulates DMC1 and RAD51 strand assimilation activity (18,20). Thus, in addition to its abrogation of the intrinsic recombinational activity of HOP2, MND1 stimulates DMC1/RAD51-mediated strand assimilation when the two proteins form the HOP2–MND1 heterodimer.

It is possible that MND1 works by inducing conformational changes in HOP2 that unmask the ability of HOP2 to stimulate DMC1/RAD51 and/or acts as a specific physical mediator between HOP2 and the recombinase. Based on these results deletion of *Mnd1* in spermatocytes most likely renders DMC1 and RAD51 inactive. In agreement with this idea, for the fraction of cells with residual expression of *Hop2* [MND1 (I)] we observed a low level of chromosome synapsis and the persistence of RAD51 and DMC1 foci through later stages of prophase I (Figure 1C and Supplementary Figure S4). We interpret these results as indicating that DSBs are created and processed followed by DMC1 and RAD51 loading on recessed DNA ends. However, in the absence of an HOP2–MND1 heterodimer initiation and/or completion of strand invasion cannot occur. Also, we cannot rule out the possibility that the absence of MND1 may result in deficient removal of DMC1/RAD51 from recombination sites.

### A dual role for HOP2 in meiotic HR

**Figure 6.**
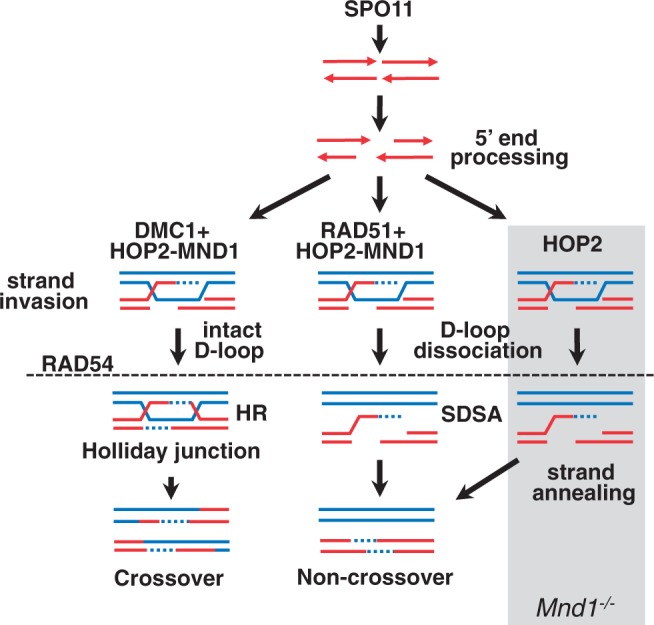
Model for suggested division of function for HOP2 in meiotic HR. HOP2 by itself repairs DSBs by catalyzing D-loops to be resolved as NCO (right branch). In a different role, HOP2 and MND1 associate to stimulate DMC1/RAD51 strand invasion. The DMC1/HOP2–MND1 cooperation results in the designation of DSBs to CO.

We propose that HOP2 has a bipartite function in mammalian meiosis (Figure 6). Our model finds support in previous work showing that HOP2 *in vitro* shows two distinctive activities: when it is incorporated into a HOP2–MND1 complex, it stimulates DMC1 and RAD51 recombination activities (14,18,20,21,23,49), and the purified HOP2 by itself is proficient in promoting strand invasion (18,20).

In this work we present *in vivo* evidence that further supports the idea that DSB repair catalyzed solely by HOP2 supports homologous chromosome pairing and synapsis. We previously showed *in vitro* that the HOP2–MND1 complex is required for efficient strand assimilation catalyzed by DMC1 and RAD51. This is in agreement with previous *in vivo* results showing that the deletion of the *Hop2* gene in mice and lower eukaryotes eliminates homologous chromosome synapsis and disrupts double-strand break (DSB) repair through HR (15–17,44,50). Therefore, assuming that absence of intact HOP2–MND1 complex will render DMC1 and RAD51 inactive, knocking out MND1 in mouse spermatocytes will result in HOP2 being the only protein with a recombinase activity available. Our observation that a fraction of *Mnd1^−/−^* spermatocytes [MND1 (II)] exhibits high level of chromosome synapsis with most of DSBs repaired (kinetics of γ-H2AX and DMC1/RAD51 are similar to wild-type) suggests that HOP2 alone promotes efficient repair of DSBs.

We also investigated the final fate of DSBs in *Mnd1^−/−^* spermatocytes. Although MND1 (II) spermatocytes reach an advanced pachytene-like stage (evaluated by histone H1T positive staining, Figure 2A), only 10% of cells showed background numbers of MLH1 foci. This indicates that events marked by MLH1 in MND1 (II) type spermatocytes that are destined to be resolved as COs are remarkably deficient. It is attractive to think that the reduced number of DMC1/RAD51 foci, which suggest repair of DSBs coupled with deficient MLH1 foci formation, indicates that most DSBs may be repaired as NCOs. Thus our results are most consistent with the view that the HOP2 recombinase activity supports a relatively advanced stage of synapsis but does not promote CO formation.

Given that the HOP2–MND1 heterodimer is essential for DMC1 function (14,18,20–23), we speculate that knocking out MND1 inactivates the pathway responsible for formation of CO (DSBR) (31).

### The molecular mechanism of recombination mediated by HOP2

Recombinases exhibit a number of biochemical properties that are considered hallmarks of homology recognition (36,51,52). Here, we investigated whether the HOP2 protein, which is proficient in catalyzing strand invasion and strand exchange, also exhibits such properties (18,20). We found that, like RecA and RecA homologs: (i) HOP2 promotes the co-aggregation of ssDNA with duplex DNA, which is known to facilitate homologous contacts; (ii) HOP2 binding to ssDNA mediates unstacking of the bases, a key step in homology recognition and (iii) HOP2 mediates the formation of a three-strand synaptic intermediate. Taken together, our results show that HOP2 shares fundamental homology-recognition properties with the RecA homologs, and unravel the molecular mechanism of recombination mediated by HOP2. This supports our model that HOP2 works as an independent *bona fide* recombinase in mouse meiosis.

A notable characteristic of HOP2 is that this protein does not belong to the RecA/RAD51/DMC1 family based on the lack of homology and on the prominent functional difference that HOP2-mediated strand assimilation reaction does not require ATP or another nucleotide cofactor [this work and (18,20)]. The reactions catalyzed by a number of recombinases from prokaryotes and eukaryotes, including bacterial RecA, RecT, Redβ and eukaryotic HOP2, RAD51, RAD52 and DMC1 differ in their requirements for an energy source for promoting homologous pairing. HOP2, RecT, Redβ and Rad52, which exhibit similar pairing activities (28,53,54), do not bind or hydrolyze ATP. DMC1 and RAD51 hydrolyze ATP weakly, whereas RecA is a robust ATPase. Even though RecA, RAD51 and DMC1 can promote homologous pairing and strand exchange in the absence of ATP hydrolysis, they require nucleotide phosphate binding (55–58). Albeit similar to RecA and the RecA eukaryote homologs HOP2 appear to enable a single strand to recognize homology in duplex DNA by a similar universal mechanism, this reaction do not require ATP.

HOP2 exhibits unique characteristics such as that it is specifically expressed in meiotic tissues and show mechanistic signatures that may distinguish it from the functions of other eukaryote recombinases. We propose a model in which HOP2 employs mechanisms of recombination that are part of a novel pathway of DNA repair that is distinct from those used by DMC1 and RAD51 (Figure 6). We observed that RAD54 efficiently dissociates D-loops formed by HOP2 (Figure 4). These results suggest that native D-loops formed by HOP2 and DMC1 are biochemically distinct and allow us to speculate that HOP2, similar to RAD51 (31), catalyzes the repair of DSBs through the formation of strand invasion intermediates that are resolved as NCO via the SDSA mechanism. In addition, the increased sensitivity to single mismatches exhibited by HOP2 compared with DMC1 suggests a possible division of function for these recombinases. It is tempting to speculate that the absence of mismatches between sister chromatids and the exquisite sensitivity to mismatches exhibited by HOP2 may suggest that this protein has a role in sister chromatid (NCO) repair.

In sum, we present data showing that HOP2 *in vivo* may work as a recombinase independently of DMC1 and RAD51. Additionally, our work with purified proteins reveals the molecular mechanisms of recombination catalyzed by HOP2. We propose that HOP2 is an ATP-independent recombinase that is part of a novel pathway of DNA repair in mouse meiosis. Based on cytological, genetic and biochemical evidence we present a model in which HOP2 plays a dual role in mammalian meiotic recombination (Figure 6). First, HOP2 alone functions as a recombinase to promote formation of strand invasion by a pathway not involving formation of CO. The possibility that HOP2 repair DSBs through the NCO pathway is a characteristic shared with RAD51 recombinase. Second, purified HOP2 interacts with MND1 to form a heterodimer that is essential for the recombinase activity of both RAD51 and DMC1. The stimulation of DMC1-mediated D-loops by HOP2–MND1 is of particular importance because it may direct the formation of CO products which are required for the proper segregation of chromosomes in meiosis.

## SUPPLEMENTARY DATA

Supplementary Data are available at NAR Online, including [59].

## FUNDING

Research reported in this publication was supported by the National Institute of Diabetes, Digestive and Kidney Diseases Intramural Research Program (to R.D.C.-O.); the National Institute of General Medical Sciences of the National Institutes of Health under award number 1P20GM103636 and by the OCAST grant [HR10-48 S to R.J.P.]; National Institutes of Health (NIH) grants [CA100839, R03DA033981, R03MH097512 to A.V.M.] and by a Leukemia and Lymphoma Society Scholar Award 1054-09 (to A.V.M.); the Russian Foundation for Basic Research (project 10-04-01057-a) (to A.A.V.). Funding for open access charge: NIH.

*Conflict of interest statement*. None declared.**

## Supplementary Material

Supplementary Data
