# Supplementary material for: The dual role of HOP2 in mammalian meiotic homologous recombination
Source: Nucleic Acids Res. 2013 Dec 3;42(4):2346–57. doi: 10.1093/nar/gkt1234 (PMC3936763; doi:10.1093/nar/gkt1234)
Supplement: Supplementary Data [file supp_42_4_2346__index.html]

The dual role of HOP2 in mammalian meiotic homologous recombination — The dual role of HOP2 in mammalian meiotic homologous recombination — Supplementary Data 

# The dual role of HOP2 in mammalian meiotic homologous recombination

## Supplementary Data

files

**Files in this Data Supplement:**

- Supplementary Data - pdf file
