# Supplementary material for: The dual role of HOP2 in mammalian meiotic homologous recombination
Source: Nucleic Acids Res. 2013 Dec 3;42(4):2346–57. doi: 10.1093/nar/gkt1234 (PMC3936763; doi:10.1093/nar/gkt1234)
Supplement: Supplementary Data [file supp_gkt1234_nar-02404-h-2013-File008.pdf]

SUPPLEMENTARY DATA

Genotyping of mice by PCR

Genotyping was carried out by PCR using oligonucleotide #17 and #18 to amplify the wild-type allele (a 400 bp fragment) and primers #17 and #19 (Table S1) to amplify the knockout allele (a 730 bp fragment). The cycling conditions were: 94°C 5 min; 94°C 30 sec, 64°C 30 sec, 72°C 30 sec for 35 cycles; 72°C 7 min.

Western blotting

For Western blot analysis, 60μg of protein from testis extracts in 20mM Tris pH 7.4, 70mM NaCl, 0.1% Triton X-100 and protease inhibitors (Roche) was separated by 4-12% SDS-PAGE and transferred to PVDF membrane (Invitrogen). Membranes were incubated with 1:500 dilution of affinity-purified anti-HOP2-MND1, and bands were detected using Oddysey infrared imaging system (LI-COR biosciences).

Real Time RT-PCR

Total RNA was isolated from adult testis with the RNeasy Mini Kit (Qiagen). 4.0µg of RNA was oligo dT primed and reverse-transcribed with Superscript II (Stratagene). The 7-8 exon boundary of *Mnd1* was amplified. Cycling conditions were as follows: 94°C 2 min; 94°C 30 sec, 55°C 45 sec, 72°C 80 sec for 35 cycles, 72°C 2 min. The 4-5 exon boundary of *Hop2* was amplified. The cycling conditions were: 94°C 2 min; 94°C 30 sec, 55°C 30 sec, 72°C 35 sec for 35 cycles; 72°C 2 min.

Histological analysis

Testes and ovaries for histological examination were removed and fixed overnight in 10% neutral-buffered formalin (Sigma). Serial sections from either testes or ovaries were positioned on microscope slides and analyzed using either hematoxylin and eosin staining or TUNEL assay (Roche). Gross and histopathologic diagnoses of somatic tissues from the knockout mice were carried out by the NIH ORS/VRP Mouse Phenotyping Service.

RNA FISH combined with immunostaining on structure preserved nuclei

RNA FISH was performed as described in Turner, et al. [1] with minor modifications. Briefly, *Hop2* genomic DNA was PCR amplified from a BAC clone, purified, and biotinylated probes were then prepared using the biotin nick translation kit (Roche). Hybridization reactions were performed using 0.4 mg/ml of labeled probe and 10mg/ml of salmon sperm DNA in 2x SSC containing 10% dextran sulfate, 50% formamide, 1mg/ml bovine serum albumin, and 2mM vanadyl ribonucleoside. Annealing was carried out in humid chambers for 12h at 37°C. Samples were then stringently washed at 42°C (three washes with 2x SSC and 50% formamide and three washes with 2x SSC) and transferred to 4x SSC containing 0.1% Tween 20. Slides were blocked in 4x SSC, 4mg/ml bovine serum albumin, and 0.001% Tween 20, for 30 min at 37°C. Probe detection was carried out using streptavidin-AF488 (1:100), followed by amplification using biotinylated antibody to streptavidin (1:150) and then one further round of streptavidin-AF488 (1:100). For each of these steps, samples were incubated for 30 min at 37°C followed by three washes for 2 min each in 4x SSC and 0.1% Tween 20. After washing, slides were rinsed in 1x PBS, crosslinked with 4% PFA and immunostained according to standard protocols.

#1	CGTAAGATGCTTTTCTGTGACTGGTGAGTACTCAACCAAGTCATTCTGAGAATA GTG-B
#2	TCCGTGTCGCCCTTATTCCCTTTTTTGCGGCATTTCCCTCTCTCTCTCTCTCTC TCCCAGAAACGCTG
#3	AAATGCCGCAAAAAAGGGAATAA
#4	F- AAATGAACATAAAGTAAATAAGTATAAGGATAATACAAAATAAGTAAATGAATAA ACATAGAAAATAAA GTAAAGGATATAAA
#5	TTTATATCCTTTACTTTATTTTCTATGTTTATTCATTTACTTATTTTGTATTATCCT TATACTTATTTACTTTATGTTCATTT-R
#6	AAATGAACATAAAGTAAATAAGTATAAGGATAATACAAAATAAGTAAATGAATAA ACATAGAAAATAAA GTAAAGGATATAAA
#7	AATTCTCATTTTACTTACCGGACGCTATTAGCAGTGGGTGAGCAAAAACAGGA AGGCAAAATGCCGCAAAAAAGGGAATAAGGGCGACACGGAAATGTTG
#8	CACTGCTAATAGCGTCCGGTAAGTAAAATGAGAATT
#9	F-GCATTCAAGAGTATCTAGCACGAGTAATGTCACG
#10	CGTGACATTACTCGTGCTAGATACTCTTGAATGC
#11	GCATTCAAGAGTATCTGGCACGAGTAATGTCACG
#12	CGTGACATTACTCGTGCCAGATACTCTTGAATGC
#13	GCATTCAGGAGTATCTGGCACGAGTGATGTCACG
#14	CGTGACATCACTCGTGCTAGATACTCCTGAATGC
#15	GCATTCAGGAGTATCTGGCACGAGTCGATGTCACG
#16	CGTGACATCACTCGTGCCAGATACTCCTGAATGC
#17	CTGGGTTCTTGAACTTTCTGTTGATAG
#18	ATACAGTCCTCTTCACATCCATGC
#19	GAGGCTTCAATAACTGTAGGTGTT

Supplementary Table I. Summary of the oligonucleotide sequences used in this work. **-B indicates: 3'-Biotin oligonucleotide; F- 5' Fluorescein oligonucleotide; R- 3' Rhodamine oligonucleotide.**

Pezza_Supplementary Fig. 1

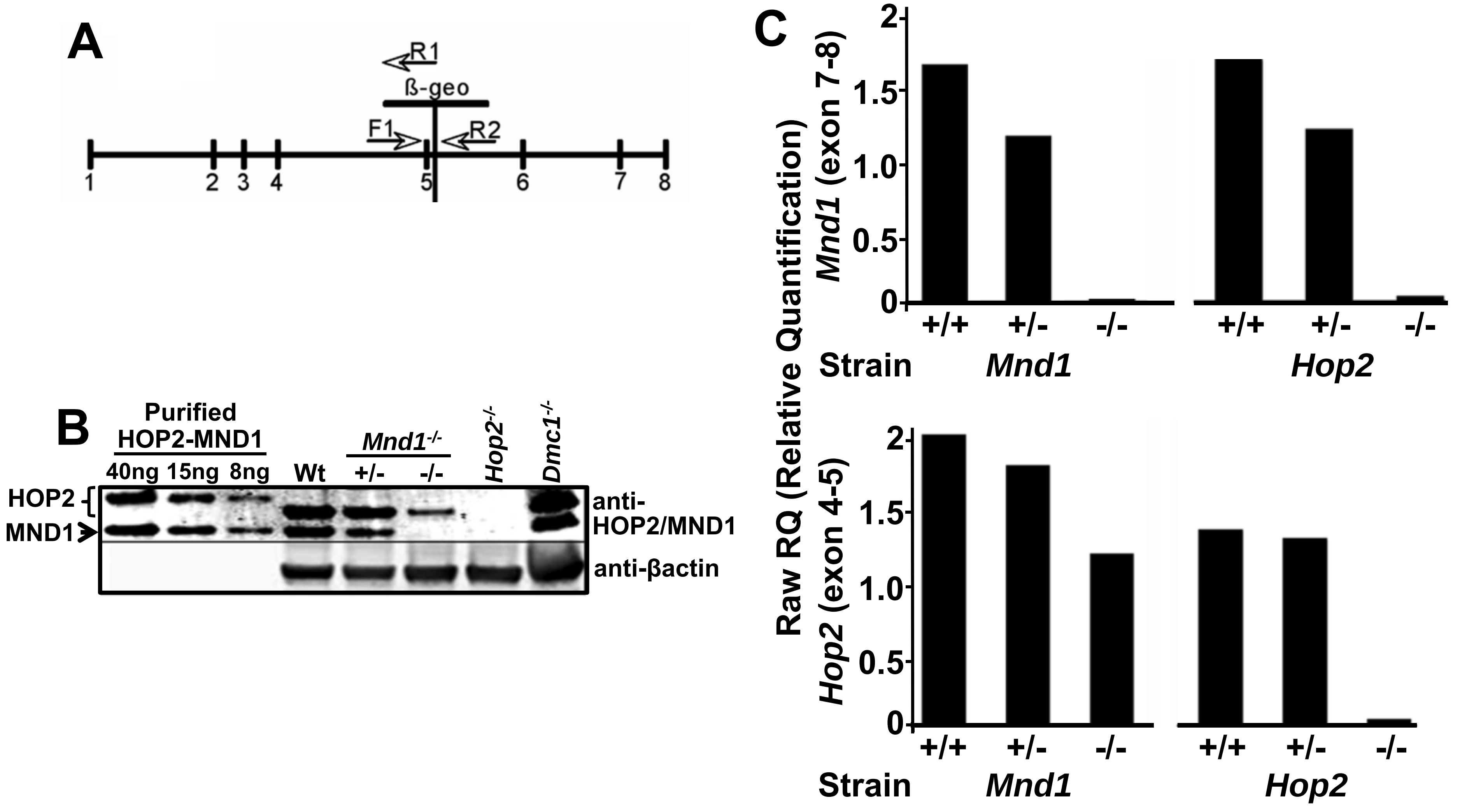

Pezza_Supplementary Fig. 2

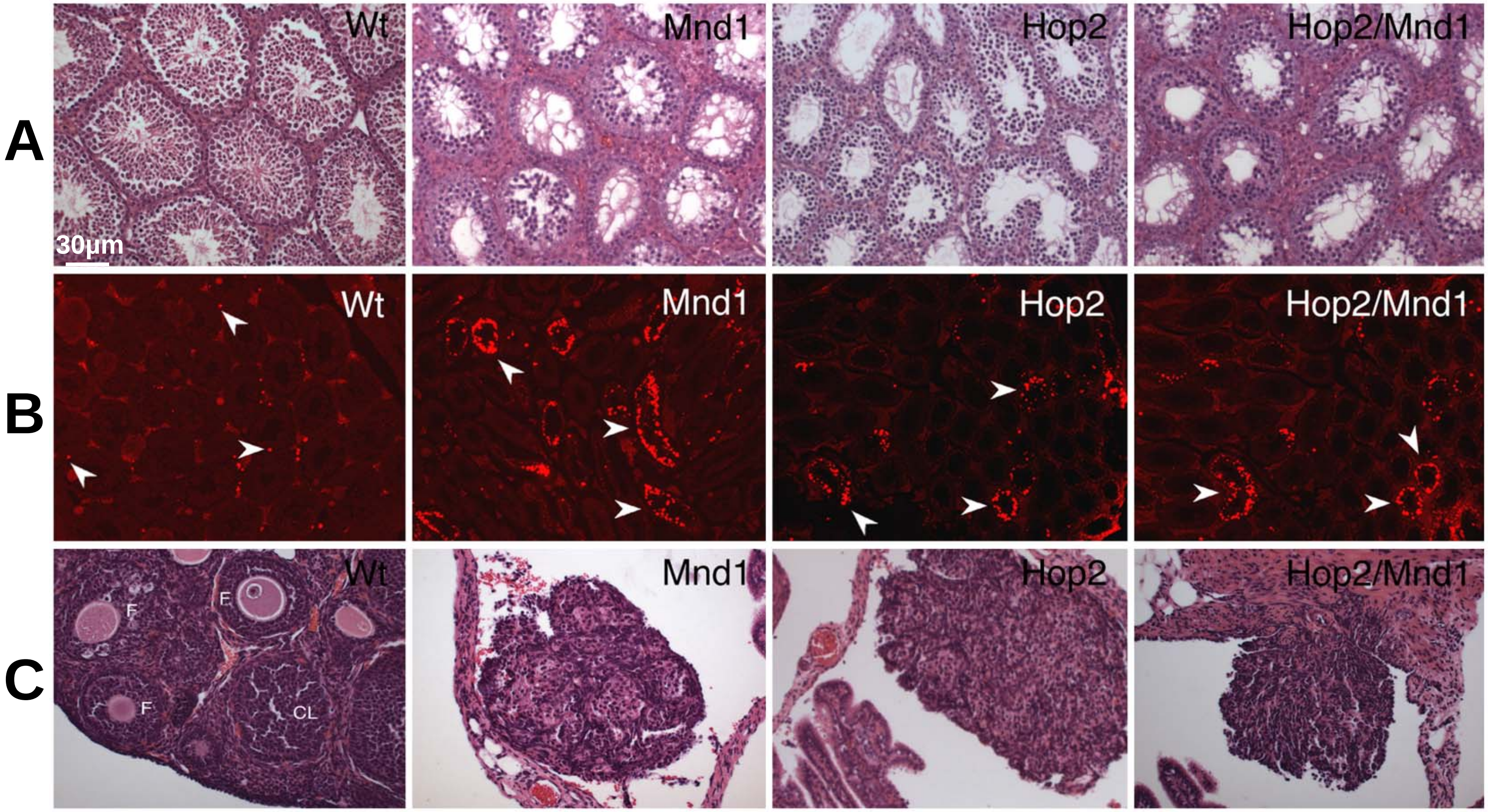

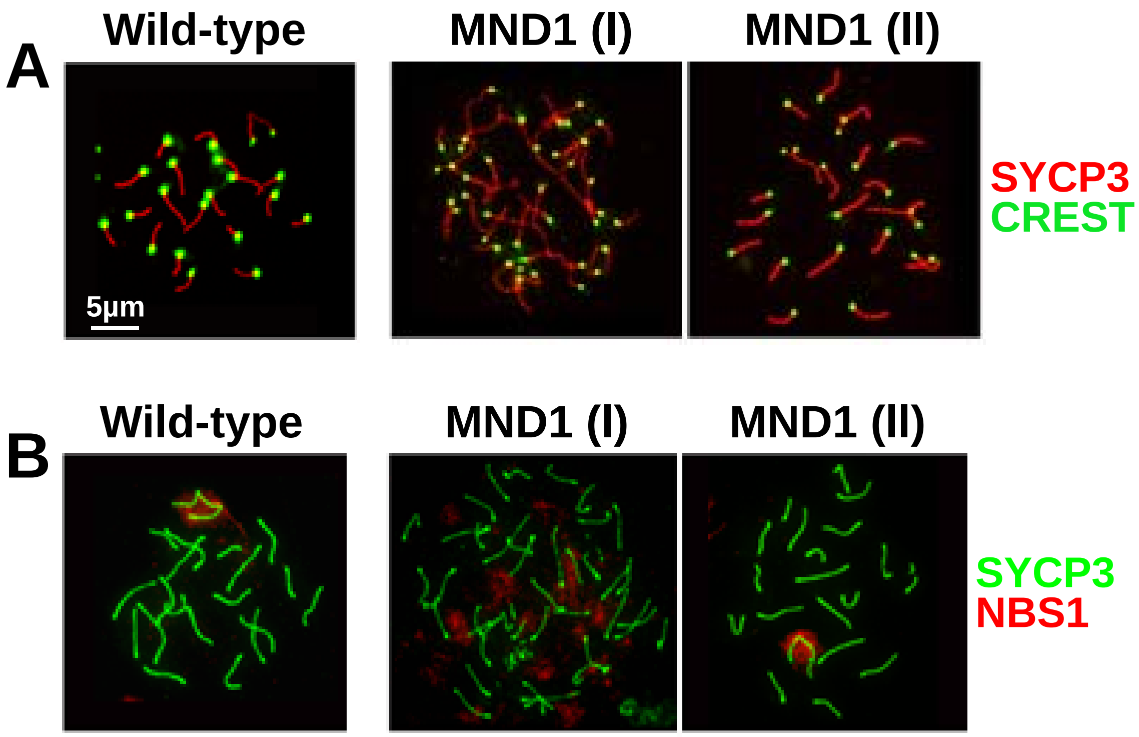
A
Wild-type
MND1 (I)
MND1 (II)
5µm
SYCP3
CREST
B
Wild-type
MND1 (I)
MND1 (II)
SYCP3
NBS1

Pezza_Supplementary Fig. 4

A

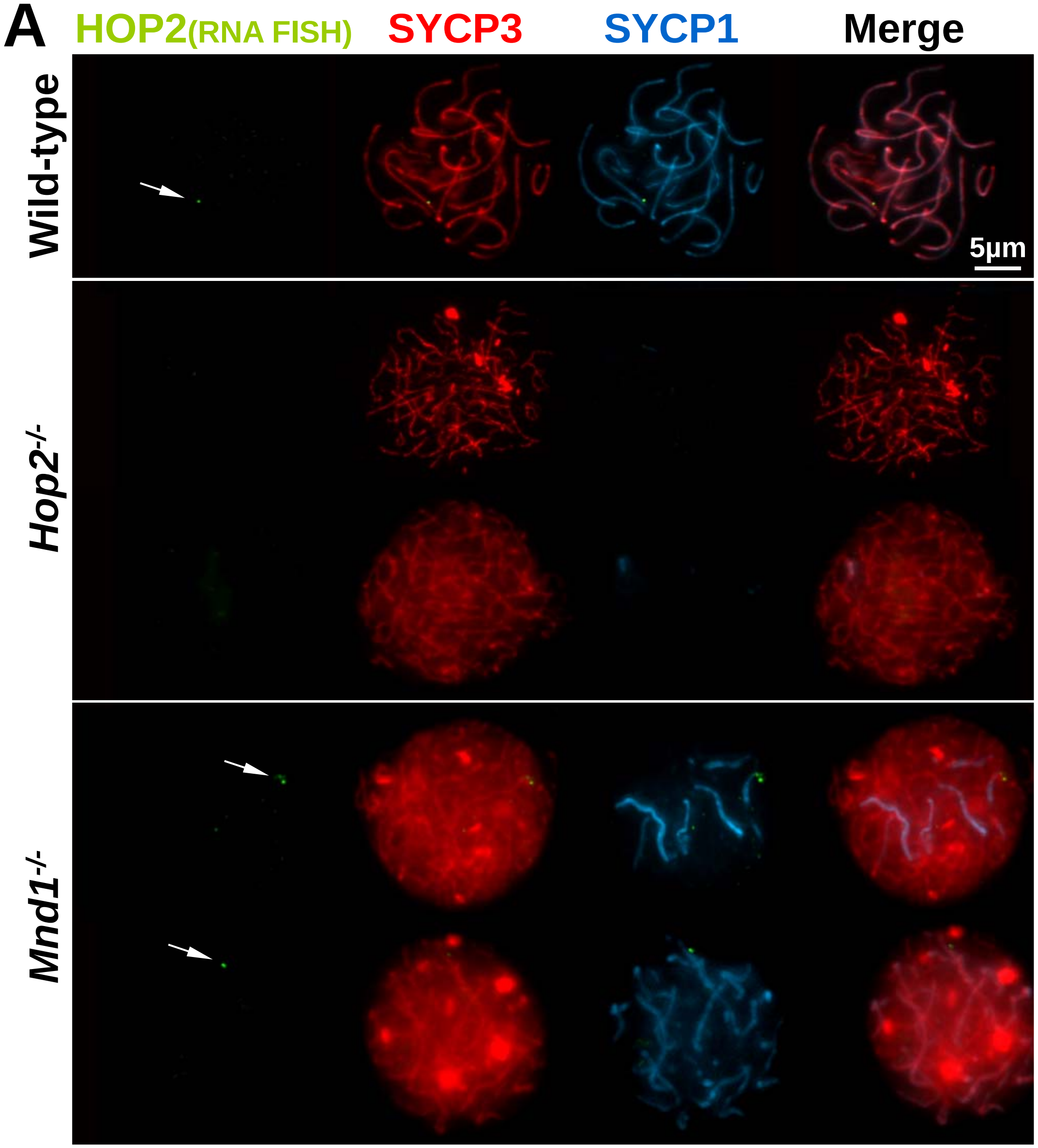

B

% of cells positive for *Hop2* transcript

Wt	*Hop2*$^{-/-}$	*Mnd1*$^{-/-}$		
60-100% synapsis	No synapsis	No synapsis	5-60% synapsis	60-100% synapsis
78% n=72	33% n=65	30% n=38	33% n=33	65% n=68

Pezza_Supplementary Fig. 5

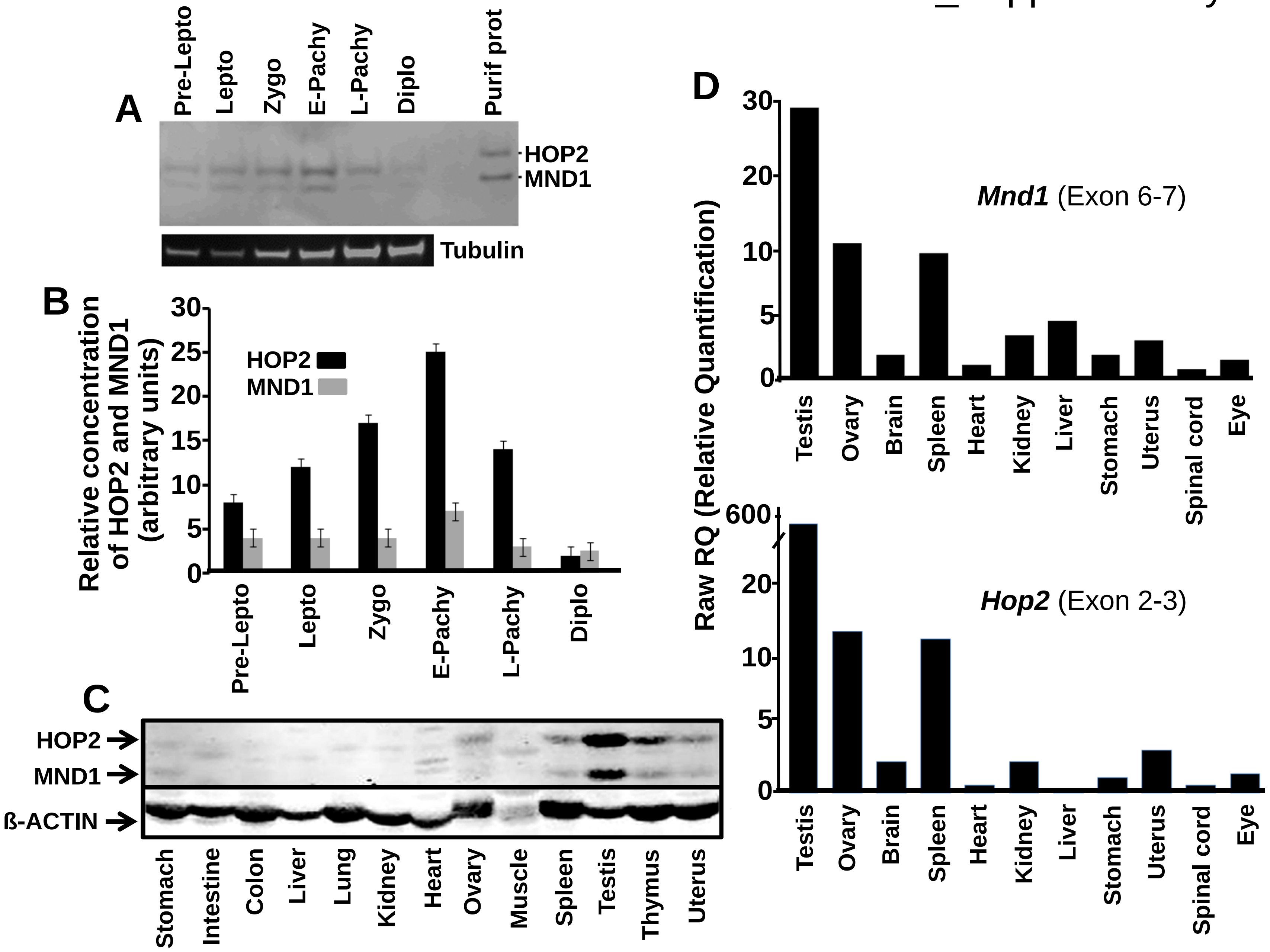

SUPPLEMENTARY FIGURES

Fig. S1. Mnd1 gene target design and expression of Mnd1 and Hop2 in wild-type and mutant mice. (A) Mouse Mnd1 gene-targeting construct. β-geo was inserted into intron 5 of Mnd1. F1/R1 and F1/R2 represent the primers used for genotyping wild-type and knockout mice, respectively. Exons are shown as numbered black bars. (B) Western blot analysis of recombinant HOP2-MND1 and testis cell extracts from wild-type, Mnd1+/- , Mnd1-/-, Hop2-/- and Dmc1-/- mice. Note the lack of MND1 and HOP2-MND1 proteins in Mnd1-/- and Hop2-/- cells, respectively. Here and in Fig. S5A purified HOP2 migrates slower compared to HOP2 from cell extracts during PAGE because it has an additional fragment derived from the expression vector at N-terminus (C) Transcript levels in testes of wild-type (+/+), Mnd1+/-, Mnd1-/-, Hop2+/- and Hop2-/- mice evaluated by real time RT-PCR.

Fig. S2. Mnd1-/- mice show profound defects in gametogenesis. (A) Histological sections of wild-type, Mnd1-/-, Hop2-/- and Hop2-/-/Mnd1-/- testes. The magnification bar represents 30 μm and corresponds to all images but shown only in one panel. (B) TUNEL assay for apoptotic cells in wild-type and knockout mouse seminiferous tubules. Note that occasional apoptotic cells are present in wild-type testes and extensive apoptosis is seen in a fraction of the seminiferous tubules of knockout mice (arrowheads). (C) Histological sections of wild-type, Mnd1-/-, Hop2-/- and Hop2-/-/Mnd1-/- knockout ovaries. Note the reduction in size and absence of folicules (F) and corpora lutea (CL) in knockout mice.

Fig. S3. Synaptonemal complex assembly defects in prophase I meiocytes from Mnd1-/- mice. (A) An example of a wild-type spermatocyte co-immunostained with SYCP3 and CREST showing complete synapsis (wild-type). Arrested Mnd1-/- spermatocytes showing no synapsis (MND1 (I)) and complete synapsis (MND1 (II)) determined by the number of CREST foci. The magnification bar represents 5 μm and corresponds to all images but shown only in one panel. (B) Pachytene-like meiocytes from Mnd1-/- mice with all asynapsed chromosomes (MND1(I)) exhibit prolonged localization of NBS1, a component of the MRE11 complex. Note that similar to wild-type spermatocytes, cells from Mnd1-/- mice with complete chromosome synapsis (MND1(II)) exhibit NBS1 staining only in the sex body region.

Fig. S4. Hop2 transcripts are detected only in Mnd1-/- spermatocytes with partially or fully synapsed chromosomes. (A) Combined RNA FISH and immunostaining were

performed on wild-type, Hop2-/- and Mnd1-/- spermatocyte nuclei structurally preserved to assess transcription in prophase I spermatocytes. Hop2 RNA FISH (green, white arrows), SYCP3 (red), SYCP1 (blue) and merged images are shown. The magnification bar represents 5 μm and corresponds to all images but shown only in one panel. (B) The extent of synapsis is based on SYCP1 staining and the percentage of cells positive for Hop2 transcripts is shown. n represents the number of scored cells. Note that only one signal for Hop2 FISH signal is observed. This indicates that chromosomes associate homologously in Mnd1-/- spermatocytes. For the wild-type sample we scored late zygotene-pachytene spermatocytes with 60-100% extent of synapsis.

Fig. S5. Expression of mouse HOP2 and MND1 proteins. (A) To assess the relative concentration of the HOP2 and MND1 proteins, wild-type spermatocytes at different stages of prophase meiosis I were isolated by cell sorting and analyzed by Western blot. (B) Quantification of signals corresponding to HOP2 and MND1 of the gel in (A) were plotted. (C) Western blot showing the MND1 and HOP2 protein pattern expression in indicated tissues of wild-type mice. (D) Real time RT-PCR amplification for Mnd1 and Hop2 of cDNA from the indicated tissues.

Supplementary References

1. Turner JM, Mahadevaiah SK, Fernandez-Capetillo O, Nussenzweig A, Xu X, et al. (2005) Silencing of unsynapsed meiotic chromosomes in the mouse. Nat Genet 37: 41-47.
